# Doxorubicin and NRG-1/erbB4-Deficiency Affect Gene Expression Profile: Involving Protein Homeostasis in Mouse

**DOI:** 10.5402/2012/745185

**Published:** 2012-08-29

**Authors:** Cecilia Vasti, Henning Witt, Matilde Said, Patricia Sorroche, Hernán García-Rivello, Patricia Ruiz-Noppinger, Cecilia M. Hertig

**Affiliations:** ^1^Instituto de Investigaciones en Ingeniería Genética y Biología Molecular—(INGEBI), Vuelta de Obligado 2490, Buenos Aires 1428, Argentina; ^2^Center for Cardiovascular Research, Charité Universitätsmedizin Berlin, Hessische Strasse 3-4, 10115 Berlin, Germany; ^3^Centro de Investigaciones Cardiovasculares, Facultad de Medicina, Universidad de La Plata, Diagonal 120 y Av. 60, 1900 La Plata, Argentina; ^4^Hospital Italiano Buenos Aires, Servicio de Anatomía Patológica y Laboratorio, Gascón 450, 1181 Buenos Aires, Argentina

## Abstract

The accumulating evidence demonstrates the essential role of neuregulin-1 signaling in the adult heart, and, moreover, indicates that an impaired neuregulin signaling exacerbates the doxorubicin-mediated cardiac toxicity. Despite this strong data, the specific cardiomyocyte targets of the active erbB2/erbB4 heterodimer remain unknown. In this paper, we examined pathways involved in cardiomyocyte damage as a result of the cardiac sensitization to anthracycline toxicity in the ventricular muscle-specific erbB4 knockout mouse. We performed morphological analyses to evaluate the ventricular remodeling and employed a cDNA microarray to assess the characteristic gene expression profile, verified data by real-time RT-PCR, and then grouped into functional categories and pathways. We confirm the upregulation of genes related to the classical signature of a hypertrophic response, implicating an erbB2-dependent mechanism in doxorubicin-treated erbB4-KO hearts. Our results indicate the remarkable downregulation of IGF-I/PI-3′ kinase pathway and extends our current knowledge by uncovering an altered ubiquitin-proteasome system leading to cardiomyocyte autophagic vacuolization.

## 1. Introduction

Overexpression of erbB2 oncogene in breast cancer cells is indicative of highly proliferative tumors with a poor prognosis following conventional chemotherapy [1]. Combined therapy of anthracycline derivatives and antibodies against erbB2 (i.e., trastuzumab, Herceptin) is clinically effective with objective tumor regressions and lower rates of both recurrence and mortality of breast cancer patients relatively resistant to tamoxifen [2, 3]. However, an undesired effect of this therapy is the severe dilated cardiomyopathy manifested in a subpopulation of treated patients. The synergistic cardiotoxicity of the combined therapy results in a 30% incidence of cardiac dilation compared to the 1–5% registered in patients receiving either trastuzumab or anthrayclines alone. Long-term retrospective analyses of trastuzumab suggest that an impaired neuregulin-1 (NRG-1) signaling sensitizes the heart towards a toxic response, that is, to anthracycline derivatives [3]. 

Murine models harboring mutations in any component of the NRG-1 signaling through tyrosine kinase receptors erbB2 and erbB4 have demonstrated that this pathway is critical for cardiac development and the maintenance of proper adult heart remodeling and function. Conditional deletion of either erbB2 or erbB4 receptors in ventricular muscle leads to dilated cardiomyopathy in adult mice [4–6]. Despite the evidence on the essential role of NRG-1 signaling in the adult heart, the specific cardiomyocyte targets of the active erbB2/erbB4 heterodimer remain unknown. 

The subcellular localization of both erbB2 and erbB4 proteins to the T-tubule membrane system [5, 6] may provide functional clues as integrators of environmental signals active in the maintenance of cardiac structure, as well this accumulation into precise sites of the cardiomyocyte membrane may facilitate the exposure to trastuzumab, contributing to the cardiotoxic effects in human patients. 

In agreement with the hypothesis that an NRG-1-deficiency provides the substrate for the aggravated doxorubicin cardiotoxicity with a net result in cardiomyocyte damage, we searched for molecular pathways which expression and activities were exacerbated in the doxorubicin-treated erbB4-KO. Therefore, this study focused on the remodeling nature and on the molecular bases of cardiomyocyte loss in the doxorubicin-treated erbB4-KO.

We employed histological and immunochemical assays to identify the morphological changes and a cDNA microarray to assess the gene expression profile in the three models of dilated cardiomyopathy utilized: ventricular muscle-specific erbB4 knockout (erbB4-KO), doxorubicin-treated wildtype (WTD), and erbB4-KO (erbB4-KOD). Gene expression data was verified by real-time RT-PCR, and then clustered into functional categories. The aggravated condition of doxorubicin-treated erbB4-KO hearts resulted in the hypertrophic enlargement of cardiac chambers, which may involve erbB2-mediated mechanisms. This study extends our current knowledge by uncovering the downregulation of IGF-I/PI3′-Kinase complex with the altered activity of the ubiquitin-proteasome system in cardiomyocytes, leading to an abnormal protein homeostasis with significant autophagic vacuolization.

## 2. Materials and Methods

### 2.1. Breeding and Analysis of erbB4 Gene-Targeted Mice

All experimental protocols were approved by the CICUAL (commission for care and use of laboratory animal) at the University of Buenos Aires, in accordance with the National Institutes of Health “Guide for the Care and Use of Laboratory Animals” (US DHHS Publication number 85-23, Revised 1996). 

The *erbB*4F/F mice C57Bl/6 were crossed into *erbB4+/−*:*MLC2v*CreKI*+/−* mice and maintained in colonies. The genotype of individual mouse was determined by PCR on tail DNA. The expression of *erbB4 *WT-, and recombined alleles was also verified in a subgroup of animals by RT-PCR (data not shown) using primers corresponding to exons 1 and 4 of *erbB4 *cDNA as previously described [6]. 

### 2.2. Doxorubicin Treatment

Isogenic, sex and age matched wildtype and knockout littermates of C57Bl/6 background were treated with doxorubicin at a final concentration of 15 mg/kg for a week. The treatment consisted of 3 intraperitoneal injections administered in 3 alternate days and left for 2 days prior to sacrifice to achieve sublethal cardiotoxicity [7, 8]. 

### 2.3. Histochemical and Morphological Analyses

Dissected hearts from male mice were washed in saline phosphate buffer. Hearts were fixed in 4% buffered paraformaldehyde and embedded in paraffin. Paraffin-embedded 5-*μ*m-thick serial tissue sections were rehydrated and stained with hematoxylin and eosin. The morphometric analysis of cardiomyocytes was performed in individual myocytes isolated using an alkaline dissociation method based on formaldehyde-fixed-ventricles treated with 12.5 N KOH for 24 hs [6]. Apoptosis was monitored by TdT-mediated dUTP nick end labeling TUNEL reaction (Intergen, Burlington, MA) in 5-*μ*m-thick serial tissue sections. Western blots were employed with antibodies against caspase 3 and anti-GAPDH (Santa Cruz Biotech, Santa Cruz, CA). Immunohistochemical analyses were performed in 5-*μ*m tissue sections by indirect immunostaining using antibodies against ubiquitin (Dako, Denmark). Cell membrane was counterstained with wheat germ agglutinin coupled to fluorescent probe 555 (Invitrogen, Carlsbad, CA) and nuclei with DAPI (Vectashield, Vector labs, Burligame, CA). Primary antibodies were detected by fluorescent labeling with biotin-coupled antibodies and streptavidin-FITC (Amersham, GE Healthcare Bioscience, NJ), as previously described [6]. Immunostained sections were analyzed by fluorescent and confocal microscopy (Zeiss, Oberkochen, Germany). Cardiomyocytes with large ubiquitin-protein aggregates were counted in 40 consecutive fields in alternate tissue sections containing both cardiac half to the base and to the apex. 

Cellular release of cardiac troponin I (cTnI) was determined in serum, utilizing a cTnI ELISA kit according to the protocol provided by the manufacture (Access Accu TnI number 33340, Beckman Coulter Diagnostic Division, CA). 

Each experiment was performed in duplicates of at least 4 different biological samples from each of the three pathological conditions and WT. Statistical significance between groups was analyzed by ANOVA and Student's *t*-test. 

### 2.4. Contractility Determination

Isolated hearts from sex matched 3-month-old WT (*n* = 4), WTD, (*n* = 4), erbB4-KO (*n* = 4) and erbB4-KOD (*n* = 4) isogenic C57/Bl6 mice, were perfused according to the Langendorff technique at constant temperature (37°C), flow (3-4 mL/min), and heart rate (360 beats/min), as previously described [9]. The basal mechanical data obtained in erbB4-KO and erbB4-KOD mice were compared to WT and WTD mice of the same littermate. The mechanical activity was assessed through an intracardiac water-filled latex balloon connected to a pressure transducer (Perceptor disponsable transducer, Namic), achieving a left ventricular end-diastolic pressure of 5–10 mmHg. Left ventricular contractile performance was evaluated from the developed pressure (LVDP) and from the half-relaxation time (*t*1/2). The response to isoproterenol (300 nM) was determined as the left ventricular developed pressure (LVDP). 

### 2.5. cDNA Microarray

For reverse transcription (Superscript III, Invitrogen) in the presence of cyanine-coupled deoxycytidine triphosphate (dCTP; Cy3 or Cy5; Amersham Bioscience), 20 *μ*g of total RNA were used in a 30-*μ*L reaction. Labeled complementary DNAs (cDNAs) were combined with 380 *μ*L TE (10 mM Tris, 1 mM ethylenediamine tetracetic acid), 20 *μ*g mouse or human Cot1 DNA and 20 *μ*g Oligo(dA), and purified with a Microcon YM-30 column (Millipore, Billerica, MA). Samples were vacuum-centrifuged and resuspended in 7.5-*μ*L nuclease-free water, 2.5-*μ*L deposition control targets (Agilent Technologies), and 15-*μ*L DIG hybridization buffer (Roche Diagnostics, Indianapolis, IN). Denatured samples (98°C, 2 min) were hybridized on cDNA microarrays (G4104A, Agilent) in a humidified chamber at 42°C for 15 h. Four technical replicates with pooled samples were carried out for each mouse line using exchanged dye-labeled RNA probes (dye-swap). After washing, microarray slides were scanned using a microarray scanner (G2565BA, Agilent) and image analysis was performed with the Feature Extraction software 7.1.1 (Agilent). Functional gene annotation, classification, and pathway analysis was performed by gene ontology terms. David software (http://david.abcc.ncifcrf.gov/) was also employed, and connecting networks was approached with ingenuity software (http://www.ingenuity.org/). The gene expression data were deposited in the Gene Expression Omnibus (GEO) database (http://www.ncbi.nlm.nih.gov/projects/geo/) as GEO accession GSE17749. 

For the identification of differentially expressed genes in the comparison of two conditions with microarrays we used the significance analysis of microarrays (SAM) [10]. The SAM statistic identifies significant changes in gene expression by performing a set of gene-specific tests. For each gene, a score is calculated on the basis of the expression change relative to the standard deviation of repeated measurements for that gene. Genes with scores greater than a threshold delta were defined as significantly deregulated. Manual adjustment of this threshold delta allows the identification of smaller or larger gene cohorts. In addition, scores for random permutations of all measurements were calculated as well and based on the defined threshold delta a false discovery rate (FDR) was estimated. 

## 3. Results

### 3.1. Murine Model for Doxorubicin-Mediated Toxicity in a Background of Impaired Neuregulin Signaling

**Figure 1:**
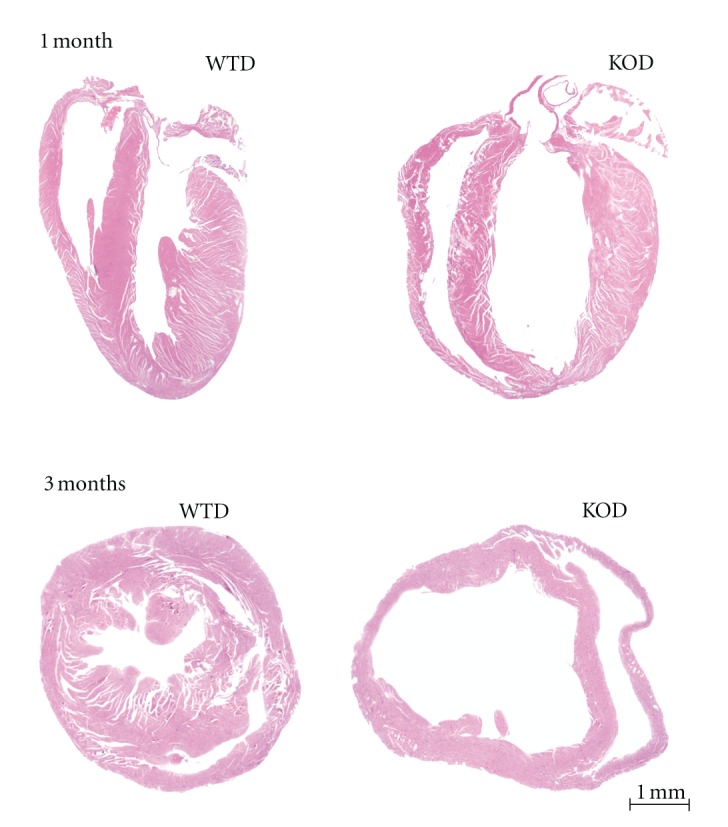
Doxorubicin exacerbates ventricular dilation in adult erbB4-KO hearts. Hematoxilin-eosin stained longitudinal and transverse ventricular sections from doxorubicin-treated (a) 1-month young adult *erbB4*F/F:*MLC2v+/+* (WTD), and *erbB4*F/F:*MLC2v*-Cre+/− (erbB4-KOD) mice and (b) 3-months adult WTD and erbB4-KOD mice display exacerbated ventricular dilation. Dilation of both RV and LV is noted in the doxorubicin-treated at the age of 1 month erbB4-KO hearts. Aggravated dilation was monitored at the age of 3 months. Scale bar: 1 mm.

The cardiac sensitization of an impaired NRG-1 signaling to the exacerbated anthracycline toxicity was comparatively assessed by doxorubicin treatment of the ventricular-specific erbB4 knockout mouse. Therefore, both wildtype (WT) and erbB4-KO mice were treated with doxorubicin (15 mg/kg), subsequently named WTD and KOD, respectively. Mice were IP injected with 3 subdoses of doxorubicin (5 mg/kg) in three alternate days within a week at two stages of postnatal development: young adults at the age of 1 month, prior to ventricular dilation, and adult erbB4-KO mice at the age of 3 months. The successive administration of doxorubicin achieved a cardiotoxicity manifested by the thinning of ventricular walls at both 1 and 3 months of age in WT and KO with the overt enlargement of the ventricular chambers in KO hearts (Figure 1). 

### 3.2. Remodeling of Cardiomyocytes

Doxorubicin mediated a significant increase in heart-to-body weight ratios in the knockouts with an average of 6.2 ± 0.8 mg/g at 1 month and of 6.7 ± 0.8 mg/g at 3 months of age with no significant differences in body weight among the three conditions and WT mice (Table 1), which is consistent with a hypertrophic remodeling. 

The cardiomyocyte remodeling was morphologically addressed by the monitoring of the cell length in KOH-isolated cardiomyocytes. In young adult mice, the doxorubicin-induced remodeling was marked by abnormally elongated cells of 137 *μ*m ± 21 *μ*m in KOD versus 100 *μ*m ± 4 *μ*m in WTD. In adult mice, doxorubicin treatment resulted in a heterogeneous cell population with 42% of abnormally elongated myocytes of 174 ± 55 *μ*m in WTD and with 65% of cells of 208 ± 65 *μ*m in KOD (Table 1). 

**Figure 2:**
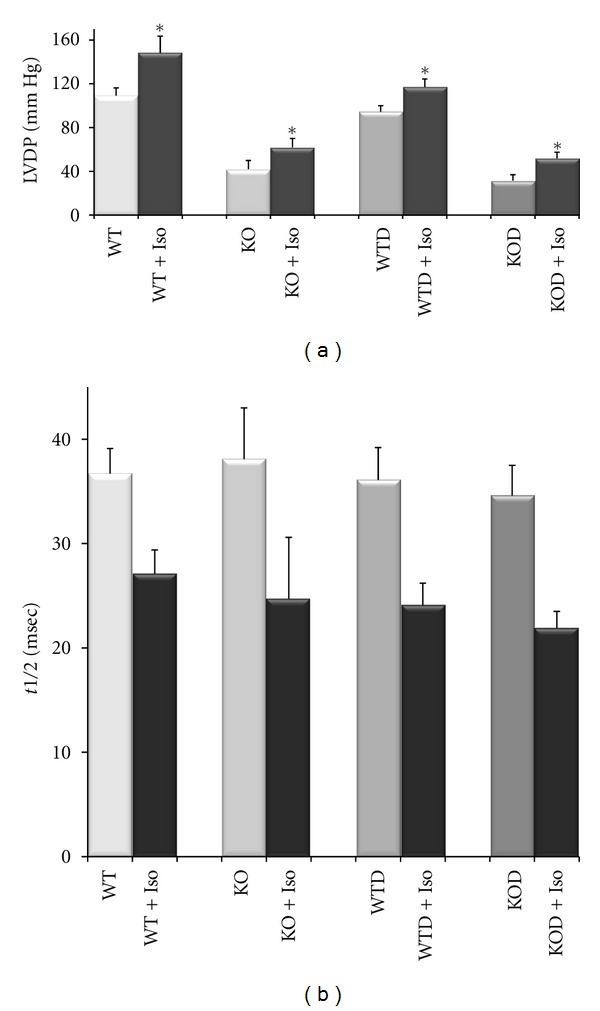
Cardiac contractility assay. Left ventricular developed pressure (LVDP, mm Hg) was determined in isolated hearts from WTD (*n* = 4) and KOD (*n* = 4) compared to WT (*n* = 5) and KO (*n* = 5) mice of both genders at the age of 3 months. Isoproterenol induced a significant increase in LVDP (a) A lower LVDP was registered in WTD and KOD relative to corresponding WT and KO. Half relaxation time (*t*1/2) was similar among the three conditions relative to WT. Values are average ± SEM, *P < 0.05.

The contractile function was determined in isolated hearts of WTD and KOD with a lower left developed pressure (LVDP) relative to the corresponding non-treated WT and KO hearts (Figure 2). The LVDP of erbB4-KOD hearts reached a 40% of WTD of the same littermate, which is similar to the nontreated KO versus Wt ratio. The inotropic response to beta-adrenergic agonists was preserved in doxorubicin-treated mice with a significant increase in LVDP (Figure 2). Doxorubicin-treated mice displayed similar and statistically significant relaxation parameters relative to nontreated mice as monitored by the half relaxation time, *t*1/2 ratios.

### 3.3. Gene Expression Profile

The remodeling of the ventricular chambers is linked to changes in gene expression involved in a panel of physiopathological conditions from hypertrophy and dilation to heart failure [11–13]. The search for differentially deregulated set of genes in dilated cardiomyopathy was performed by comparing the transcript levels from adult erbB4-KO, WTD and KOD relative to WT mouse hearts in hybridization experiments in microarrays containing over 8,500 mouse cDNA clones. 

**Figure 3:**
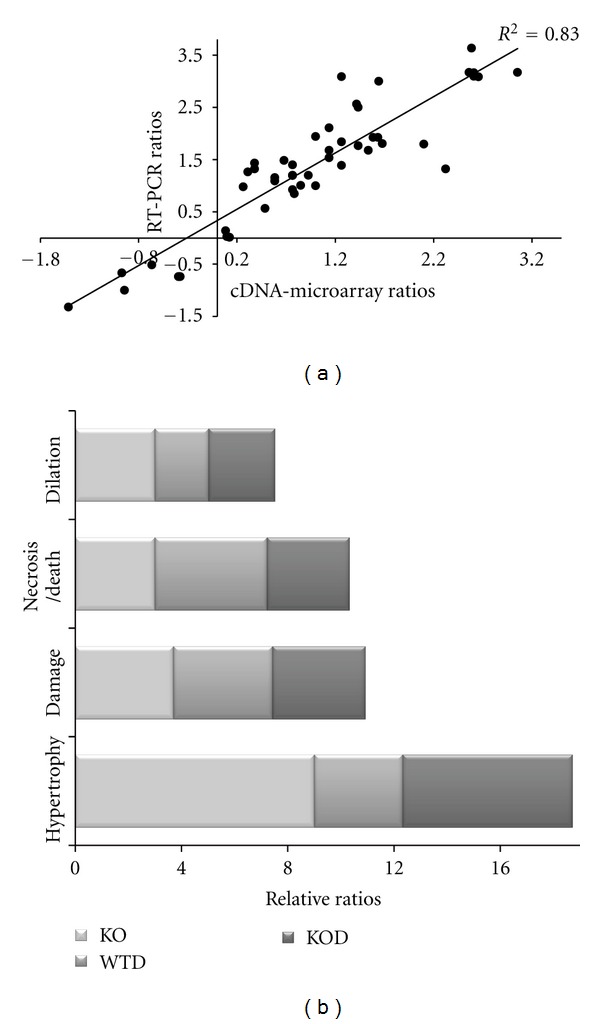
Validation of cDNA microarray ratios and functional clustering. The cDNA array ratios were correlated to RT-PCR ratios of differentially expressed genes. (a) Following logarithmic transformation, RT-PCR ratios were plotted against cDNA array ratios. (b) Differentially expressed genes among erbB4-KO, WTD, and erbB4-KOD hearts using the criteria of FDR < 10 were enriched in GO terms and functionally clustered. The graphics represent top groups enriched in number of genes and expression fold change corresponding to cardiac disorders for KO, WTD, and KOD hearts relative to WT.

The significant expression changes in set of genes identified in the three mouse conditions by cDNA microarray data analysis was verified with quantitative RT-PCR (Figure 3) in mRNA samples from male and female ventricles of 1 and 3 months of age. The gene expression level determined by quantitative RT-PCR (qRT-PCR) within individual samples was normalized against GAPDH mRNA, and compared to microarray ratios. The high consistency in the correlation of the array value ratios and the qRT-PCR ratios (r : 0.83) demonstrated the high fidelity of the microarray analysis to detect gene expression changes (Figure 3(a)). 

To interpret the list of genes, we performed a computational analysis for enrichment of gene ontology (GO) terms [14]. Among the top functionally toxic or disease groups, a large set of genes were associated to hypertrophy, necrosis/cell death, cardiac damage, and dilation. The hypertrophic group of genes prevailed in the knockouts (KO, KOD) over other classifications, this group was less represented in WTD hearts, which was underlined by a general cell death group (Figure 3(b)). 

To investigate molecular targets and pathways implicated in the exacerbated cardiotoxic phenotype, we performed gene clustering and pathway analyses to get insight into severely modified functional groups that may account for the sensitize condition of erbB4-KO hearts to doxorubicin toxicity. 

### 3.4. Commonality in Gene Expression Profile

#### 3.4.1. Hypertrophy-Related Genes

The heart of all three mouse models showed the classical signature of a hypertrophic response by the upregulation of BNP (*Nppb*), CARP (*Ankrp*), skeletal muscle-actin, *c-fos*, LIM domain proteins, and cyclin-dependent kinase inhibitor 1A, p21 (*cdkn1a*). However, a hallmark of hypertrophy-related genes, the atrial natriuretic peptide (*Nppa*), was found slightly increased in WTD hearts compared to the high ratios determined in the KO, with intermediate ratios in KOD (Table 2). 

#### 3.4.2. Hypertrophy-Related Mechanisms

The functional clustering of large set of genes allowed for the search of mechanisms leading to hypertrophy. This analysis positioned a cohort of mediators, including the upregulation of heparin binding-epidermal growth factor (*Hb-egf*), *cdkn1a*, *Ankrp*, Serum/glucocorticoid kinase (*Srgk*), G protein-coupled receptor kinase 5 (*Gprck5*), and *Gadd45g, *in an erbB2-dependent hypertrophic signaling (Table 2), with a higher number of significantly deregulated genes in KOD relative to WTD and KO. 

### 3.5. Cell Cycle in Hypertrophic Remodeling

The reduction in cell proliferation in the three conditions was indicated by deregulation of cell-cycle-associated genes (Table 2). The decreased level of mitosin, cyclins, and cyclin-dependent kinase messenger RNA were accompanied by the increased expression of the antiproliferative cyclin-dependent kinase inhibitor 1A (p21), also upregulated in other models of cardiomyopathy [11, 12]. 

### 3.6. Cell Survival and Apoptosis

**Figure 4:**
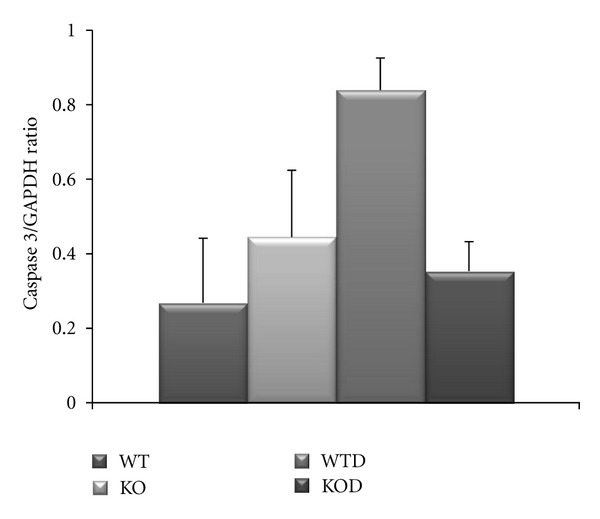
Cellular level of Caspase 3. The active caspase 3 (17 kD) level was determined by Western blots from ventricular extracts of 1 and 3 months treated and nontreated WT and KO and normalized against GAPDH. The caspase 3/GAPDH ratios were plotted as average ± SD (*n* = 4).

Cell survival and stress-related genes *Bcl-2 *and Bcl-2-like (*Bcl2l1*) were slightly upregulated in the knockouts, with no changes in pro-apoptotic genes (*Bax, Fas, FasL*) as monitored by qRT-PCR. The expression of apoptotic-related genes, that is, *Gadd45g *and B cell translocation (*Bcl2l1*), reached significance in doxorubicin-treated animals (Table 2). Therefore, cellular apoptosis was comparatively examined by TUNEL reaction in cardiac sections from mice of 1 month and 3 months of age. Increased cell death was observed in doxorubicin-treated hearts with an average of 0.35 ± 0.2 TUNEL(+) nuclei per field of 0.2 mm^2^, which was similar in WTD and KOD (Figure S1, Supplementary Material, available at doi:10.5402/2012/745185), apoptosis was rare or nil in nontreated WT and KO [6]. The presence of the active Caspase 3 protein was monitored in whole ventricular extracts by western blotting (Figure  S1 supplemental), showing a significant increased ratio to GAPDH in WTD samples, yet, at a low level relative to a positive control of staurosporin-treated cardiomyocytes in culture. The cellular apoptosis was therefore significant in WTD hearts as monitored by the higher expression of apoptotic-related genes, TUNEL(+) cells and the significant increase protein level of caspase 3 (Figure 4). Consistent with the induced apoptosis in WTD, genes related to oxidative stress (*Nfkbia, Atf4, Map3k6, Mnk2, Mkp, Dnaja1, Gadd45g*) were significantly represented in WTD hearts (Table SI, supplemental). The expression of heat shock protein 70 (*hsp1a*) reached the highest level in WTD, in agreement with oxidative stress conditions [15]. 

### 3.7. Gene Expression Profile in Exacerbated Cytotoxicity

#### 3.7.1. Growth Factor Expression Profile

A deregulated cytokine profile was significant in the erbB4-KOD, which included the downregulation of natriuretic peptide receptor, angiotensin-like receptor and the upregulation of IL-2 associated to stress and inflammatory responses [16]. Most remarkable was the significant imbalanced expression of a cohort of key cardiac growth factors (e.g., *Egf, Hb-egf, Ptn, Posn, Gcdnf, Fgf1, Igf-I*) that underlined the erbB4-KOD (Table 3). The overexpression of *Hb-egf *was statistically significant in WTD and KOD mice, with a significant upregulation of *Egf *in KOD (Table 3), associated to pathological forms of hypertrophy [13, 17, 18]. In contrast, the insulin-like growth factor-I (IGF-I), implicated in physiological hypertrophy [19], was downregulated in the knockouts, exhibiting a severe decay in erbB4-KOD with the compromised expression of molecules of the PI3′-kinase complex (Table 3). Growth factors regulated by IGF-I, that is, hepatic growth factor (*Hgf*) and vascular endothelial growth factor C (*Vegfc*) exhibited a significant 40% decay. An additional list contains other highly significantly up or down regulated genes of Wt versus erbB4-KOD, not discussed in this manuscript (Table SII, supplemental).

#### 3.7.2. Ubiquitin-Proteasome System

**Figure 5:**
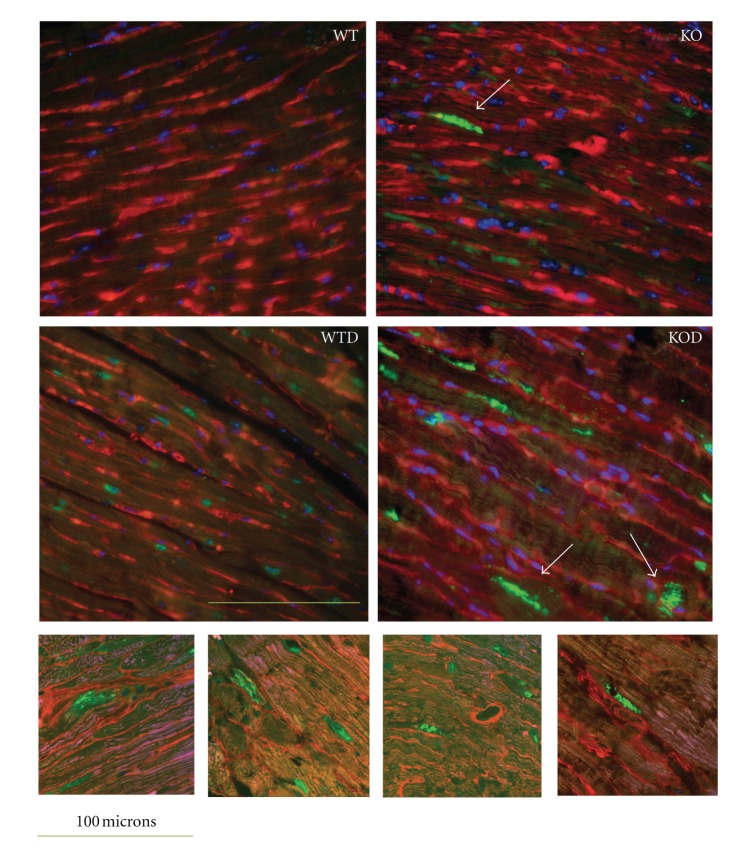
Protein ubiquination in cardiomyocytes. Transverse ventricular sections of nontreated WT and KO and doxorubicin-treated WT and KO hearts (WTD, KOD) (*n* = 4) were immunnostained with antibodies against ubiquitin, revealed with biotin-coupled antiolyclonal rabbit and FITC-coupled streptavidin (green). The membrane was stained with wheat germ agglutinin (TRICT-coupled WGA, red) and nuclei with DAPI (blue). The images were scanned with fluorescent microscope (upper panel), original magnification 400x. Scale bar: 100 *μ*m. Note ubiquitin-protein aggregates (arrows) and ubiquitin-labeled proteins adjacent to the membrane in erbB4-KOD. Detailed images of KOD sections with large ubiquitin-posistive protein aggregates were obtained by confocal microscopy (lower panel).

**Figure 6:**
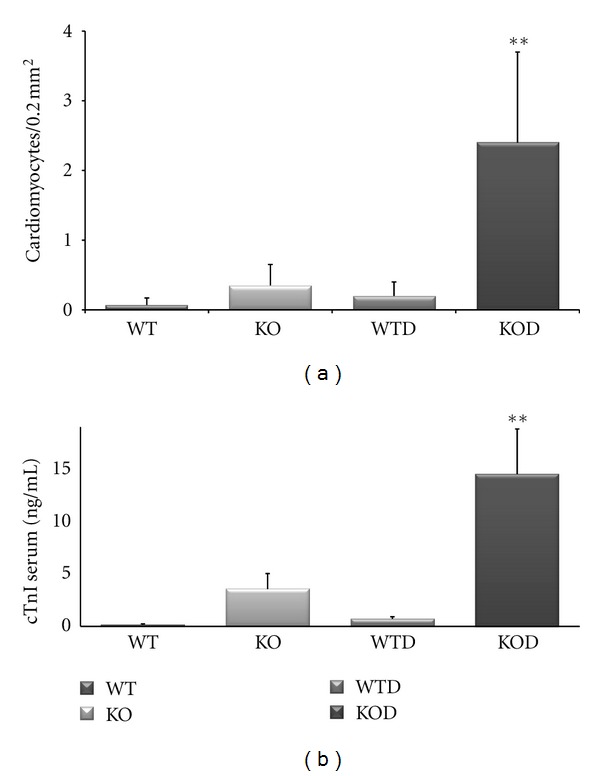
Cardiomyocyte Autophagic Vacuolization and release of cTnI. (a) Cardiomyocytes exhibiting large ubiquitinated protein aggregates (arrow) from images as shown in Figure 5 were counted in consecutive fields of 0.2 mm^2^ from cardiac section of nontreated and doxorubicin-treated 1 month and 3 months WT and KO hearts (WTD, KOD) (*n* = 4). The average values ± SD were plotted with a significant number of ubiquitin (+) cardiomyocytes in KOD, **P < 0.01. (b) The release of cTnI was determined in whole serum from WT, KO, WTD, and KOD mice (*n* = 4), the average values ± SD were plotted (*n* = 4), **P < 0.001.

Concurrent to a requirement for the local activity of IGF-I in the prevention of ubiquitin-mediated muscle atrophy [20], the decay of IGF-I in erbB4-KOD is accompanied by the deregulation of a relevant set of genes of the ubiquitin-proteasome system in erbB4-KOD. The remarkable upregulation of inducible related-ligase genes and the deregulation (up or down) of associated proteases (Table 4) in erbB4-KOD hearts may result in an abnormal protein homeostasis. To evaluate the biological incidence of the deregulated ubiquitin-proteasome system components in KOD cardiomyocytes, we performed immunostaining assays by employing antibodies against ubiquitin in cardiac sections from mice at the age of 1 and 3 months (Figure 5). Ubiquitin (+) protein aggregates were found exacerbated in KOD cardiomyocytes with an average of 2.4 ± 1.3 cardiomyocytes/0.2 mm^2^ versus 0.35 ± 0.3/0.2 mm^2^ in KO and 0.12 ± 0.08/0.2 mm^2^ in WTD (Figure 6(a)). The potential cardiomyocyte damage as a net result of the aggravated doxorubicin toxicity in the erbB4-KOD was monitored by immunodetection of cardiac troponin I (cTnI) serum levels. A remarkable increase in cTnI was determined in KOD with an average of 14.5 ± 4.2 ng/mL, with intermediate levels in WTD and KO and relative to the low or nil level in WT (Figure 6(b)). 

Taken together, these results reveal a severe deregulation of the ubiquitin-proteasome system with excessive protein aggregation and cardiomyocyte damage with release of cTnI, which underline the aggravated doxorubicin-toxicity in erbB4-KOD hearts. 

## 4. Discussion

Relevant evidence demonstrated the essential role of NRG-1 signaling through cognate erbB2/erbB4 heterodimers in the heart, moreover, clinical data has revealed that an impaired NRG-1 signaling potentiates the cardiotoxic effect of anthracycline therapies. In this study, we examined severely affected pathways in the ventricular-specific erbB4-KO at the age of 1 and 3 months following doxorubicin treatment that may account for the exacerbated cardiotoxicity.

### 4.1. Hypertrophic Response to NRG-1/erbB4-Deficiency and Doxorrubicin

Among major overexpressed group of genes, the hypertrophy-related components were a commonality among the three mouse conditions. Compared to WTD hearts, characterized by a thinning of ventricular walls with an unchanged heart-to-body weight ratio, the KOD manifested an increase in the heart-to-body weight ratio accompanied by an intermediate ANF level relative to the highest and lowest ratios determined in the KO and WTD, respectively. The erbB2-dependent mechanism was represented by a large number of significantly deregulated genes in erbB4-KOD, suggesting the contribution of this pathway to the enlargement of the heart. 

### 4.2. Cellular Apoptosis

The significant upregulation of apoptotic-related genes together with the overexpression of oxidative stress pathway components underlined the heart condition of WTD mouse, which is in agreement with published evidence [21, 22]. In WTD hearts, apoptotic mechanisms were also found significantly active as monitored by the increased level of active caspase 3 and the presence of TUNEL(+) nuclei in cardiac sections. The KOD manifested a similar level of TUNEL(+) cells relative to WTD instead of an exacerbated mitochondrial-dependent toxicity that could account for the aggravated cardiac remodeling of KOD heart. 

### 4.3. Growth Factor Disorder in KOD

In this study, we have established a cohort of relevant growth factors, whose imbalanced expression maybe overall associated to cardiac remodeling disorders. In erbB4-KOD hearts, the severe decay of IGF-I, required for physiological hypertrophic remodeling, likely converge into the remarkable dilation and thinning of ventricular walls with heterogeneous cardiomyocyte population of an abnormal length. Relative to adult KOD, the decay in the IGF-I mRNA level as determined by qRT-PCR was found similarly low in KOD at 1 month of age. In this regard, the IGF-I expression profile maybe relevant to NRG-1 impaired activities in remodeling rather than to a general cardiac function deficit. 

In addition to the individual activities as powerful enhancers of muscle growth, both NRG-1 and IGF-I have been proven to synergize activities for both cardiomyocyte proliferation and myofibrillogenesis through PI3′-kinse/Akt and Ras-MAPK pathways respectively, in ex vivo models and in cultured cells [23]. In this context, it is suggested that growth factor interactions may also modulate the dominant proliferative or differentiating activities of the factors per se. 

In a similar setting to erbB4-KOD mice, the induced doxorubicin-cardiotoxicity in NRG-1 heterozygous mice leads to a decreased activation of Akt and MAPK pathways [24]. 

### 4.4. Ubiquitin-Proteasome System Deregulation Mediates Homeostasis Block in KOD

Recently, a role for PPAR*α*/IGF-I, downregulated in KOD, has been suggested to conform a cardioprotective axis against cell damage during ischemia and hemodynamic conditions [25], as well local IGF-I activity was demonstrated to prevent the activation of the ubiquitin-proteasome pathway in failing human hearts [20, 26]. In the erbB4-KOD, components of the ubiquitin-proteasome system were found severely deregulated with the overexpression of critical regulators, such as inducible ubiquitin-ligase enzymes [27], and the up- or downregulation of related proteases. In this regard, the deregulation of the ubiquitin-proteasome system, likely linked to the decay in IGF-I/PI3′-kinase, represents a main functional group synergistically affected in the erbB4-KOD heart.

The number of cardiomyoctes affected by large protein aggregates in erbB4-KOD represented a 7-fold increase relative to the number of erbB4-KO cardiomyocytes with a similar ubiquitinated profile, indicating excessive autophagic vacuolization in KOD. In this regard, protein ubiquitination is recognized to exert a dual role in both cardioprotection and cell death mechanisms [28–31]. Autophagic cell death was revealed by the appearance of large ubiquitin-positive protein aggregates [30, 31], named autophagic vacuolization according to recent nomenclature recommendations [32]. Autophagic vacuolization has been also suggested to be useful predictor of myocardial deterioration [33]. 

In KOD mice, cardiomyocyte damage was indicated by the significant increase in serum levels of cardiac troponin I. Interestingly, this result agrees with human patient data suggesting that cTnI serum level is a sensitive cardiotoxic marker for patients following trastuzumab and doxorubicin therapy [34, 35]. 

In summary, these results extend our current knowledge by demonstrating that the response of erbB4-KO heart to doxorubicin toxicity has a net result in excessive autophagic vacuolization, consistent with the association of an abnormal protein homeostasis with severe cardiac disorders. 

## Figures and Tables

**Table 1:** Doxorubicin-mediated cardiomyocyte phenotypic modifications. Heart weight and body weight were monitored in WT, WTD, erbB4-KO, and KOD (*n* = 20) at 1 and 3 months of age. Hypertrophic growth is indicated by the increase in heart-body-weight ratio in erbB4-KO and KOD compared to WT and WTD. Cell length was determined in isolated cardiomyocytes from hearts of 1 and 3 months of age (*n* = 4). The length average was increased in young adult KOD. The average over 129 *μ*m long was increased in KO, WTD, and KOD compared to WT hearts. Values are means ± SD; Data changes were statistically compared to WT, ^#^ *P* < 0.05; *P < 0.01.

Morphology	WT	KO	WTD	KOD
Young Adult (1 month)				
Heart/Body weight (mg/g)	5.2 ± 0.4	5.3 ± 0.6	5.2 ± 0.5	6.2 ± 0.8^#^
Body weight (g)	17.4 ± 2.0	17.3 ± 2.2	17.3 ± 2.0	17.2 ± 1.5
Average cell length > 90 *μ*m	95 ± 10	102 ± 8	100 ± 4	137 ± 21^#^
Adult (3 month)				
Heart/Body weight (mg/g)	5.6 ± 0.6	7.3 ± 0.8*	5.4 ± 0.7	6.7 ± 0.8
Body weight (g)	30.1 ± 2.5	30.3 ± 2.2	29.4 ± 2.2	28.4 ± 2.2
Average cell length > 129 *μ*m, (%)	152 ± 14 (15)	195 ± 50* (75)	174 ± 57^#^ (42)	208 ± 58* (65)

*P < 0.01, ^#^ *P* < 0.05.

**Table 2:** Commonality in set of genes related to hypertrophy and cell cycle. Differentially expressed set of genes verified by real-time RT-PCR. Representative genes associated with hypertrophy, and related to erbB2-dependent hypertrophic signaling, were found significantly upregulated in the three conditions. Cell cycle genes were preferentially downregulated with overexpression of *Cdck1a*. Data was normalized to glyceraldehyde 3-phosphate dehydrogenase (*Gapdh*) expression and to WT. Data are expressed as mean value ratios at FDR < 10 significance, ns: not significant.

Gene	ErbB4-KO		WTD		ErbB4-KOD	
	Ratio	FDR	Ratio	FDR	Ratio	FDR
Hypertrophy						
actin, alpha, skeletal muscle (acta1)	2.7	1.7	1.2	ns	1.7	5.4
cardiac ankyrin repeat protein (Carp)	6.1	1.7	2	0.6	2.4	1.5
matrix gamma carboxyglutamate protein (mglap)	2.2	5.1	1.5	3.9	1.7	1.5
four and a half LIM domains 1 (fhl1)	3.3	1.7	1.8	1.3	6	6.1
natriuretic peptide precursor A (nppa)	5.9	1.7	1.4	7.8	2.9	3.3
natriuretic peptide precursor B (nppb)	6.1	1.7	8.3	0.6	6.3	1.5
cardiomyopathy associated 1 (Cmya1)	1.1	ns	3.1	0.6	2.4	2
Hypertrophic erbB2-signaling						
Heparin binding epidermal growth factor (Hbegf)	1.4	n.s	4.6	0.6	4.5	14.7
Fibrinogen-like protein 2 (Fgl2)	2.0	1.6	1.0	ns	6.0	0.1
Cyclin-dependent kinase inhibitor 1A (cdnk1a)	2.3	1.6	3.0	0.6	2.7	0.1
Ankyrin-like repeat protein (Ankrd)	6.1	1.7	2.0	0.6	2.6	0.1
Myelocytomatosis oncogene (Myc)	1.5	21.1	2.9	0.6	1.7	0.2
FBJ osteosarcoma oncogene (Fos)	2.0	1.7	5.6	0.6	1.5	0.5
Neuregulin 4 (Nrg4)	1.8	n.s.	1.4	9.1	1.3	0.2
Epidermal growth factor (Egf)	0.6	5.1	1.3	ns	4.6	0.6
Serum/glucocorticoid regulated kinase (Sgk)	1.5	n.s.	2.2	0.6	1.9	0.1
G protein-coupled receptor kinase 5 (Grk5)	1.5	n.s.	0.9	ns	2.9	0.1
Cell cycle						
emerin	0.6	4.2	1.1	0.6	0.5	3.5
cyclin-dependent kinase inhibitor 1A p21 (cdkn1a)	2.3	1.7	3	0.6	2.7	2
B-cell translocation gene 2, anti-proliferative (Btg2)	1.3	ns	2.2	0.6	1.6	7
BCL2-like (Bcl2I1)	1.7	1.7	3.2	0.6	2.2	2.7
growth arrest and DNA-damage-inducible 45 *γ* (Gadd45g)	1.3	ns	1.7	0.6	1.9	1.5

FDR: false discovery rate (%), FDR > 10 = not significant (ns).

**Table 3:** Deregulated expression of growth factor and IGF-I pathway genes. Deregulated expression of growth factor genes underlines erbB4-KOD hearts by the upregulation of *Egf, Hb-egf, Ptn*, *Post *and downregulation of *Fgf1 *and *IgfI*. Comparative expression of genes related to the IGF-I pathway exhibits an extended downregulation in erbB4-KOD relative to KO hearts. Abbreviations: phosphatidylinositol 3-hydroxi kinase (PI3′-K); murine thymoma viral oncogene homolog 2 serine/threonine protein kinase (Akt2). Statistically significant data correspond to fold change ratios at FDR < 10, ns: not significant.

Gene	Genebank	ErbB4-KO		WTD		ErbB4-KOD	
		ratio	FDR	ratio	FDR	ratio	FDR
Growth factors							
Fibroblast growth factor 1 (Fgf1)	AA261582	0.6	4.9	1.0	ns	0.7	14
Glial cell derived neurotrophic factor (Gdnf)	W45748	1.3	ns	1.8	5.3	1.3	ns
Gndf receptor 3 (Gdnra3)	AA050083	2.0	2.0	1.1	ns	1.6	2.0
Epidermal growth factor (Egf)	AI326499	0.6	5.0	1.3	ns	4.6	0.6
Egf-fibulin-like extracellular matrix (Efemp)	AI156278	0.7	9.5	0.7	ns	6.4	8.9
Heparin binding-Egf (Hbegf)	AI595201	1.4	ns	4.6	0.6	4.5	9.4
Pleiotrophin (Ptn)	AI047160	1.0	ns	2.6	ns	4.5	0.5
Secreted frizzled-related (Srfp)	AI019575	1.0	ns	1.2	ns	2.0	0.7
Periostin, osteoblastic factor (Postn)	W81878	1.5	3.5	0.9	ns	1.5	1.5
Insulin-like growth factor-I (Igf1)	AI604642	0.7	ns	1.1	ns	0.35	0.2
IGF-I signaling							
Insulin-like growth factor-I (Igf1)	AA822429	0.75	16	1.1	ns	0.52	0.2
Insulin-like growth factor-I (Igf1)	AI604642	0.74	15	1.1	ns	0.35	0.1
Igf1 binding protein 1 (Igfbp1)	AI892189	1.2	n.s.	1.0	ns	0.55	0.1
PI 3′K regulatory subunit p85 beta (Pik3r1)	AA033042	0.8	n.s.	0.8	ns	0.32	0.1
serine/threonine protein kinase (Akt2)	AA437947	0.7	5.07	0.9	ns	0.58	0.1

FDR: false discovery rate (%), FDR > 10 = not significant (ns).

**Table 4:** Deregulation of ubiquitin-proteasome system genes. Comparative expression levels among the three models. Upregulation of Ubiquitin ligase, associated regulatory genes and deregulation of related proteases are significant in erbB4-KOD. Significant data correspond to fold change ratios at FDR < 10, ns: not significant.

Gene	Genebank	ErbB4-KO		WTD		ErbB4-KOD	
		ratio	FDR	ratio	FDR	ratio	FDR
Ubiquitin-Proteasome system							
Ubiquitin-conjugating enzyme 5 (Ubc5 homolog)	AA517487	1.1	ns	0.9	ns	5.6	0.5
Casitas B-lineage lymphoma b (Cblb)	AI430293	1.0	ns	1.8	0.9	2.2	2.1
Autophagy-related 4A-like (Atg4a homolog)	AI048074	0.9	ns	1.0	ns	2.0	0.2
LON peptidase (lonp)	AA241939	0.8	ns	1.6	9.1	2.0	0.5
Ubiquitin ligase complex component (Ubr)	AA615286	1.5	6.9	2.2	0.6	1.8	1.7
Carboxypeptidase X 2 M14 family (Cpxm2)	AA690480	1.4	ns	1.6	0.9	1.7	0.8
Ubiquitin-regulatory protein (Nsfl1c)	AW2095992	0.7	4.9	0.9	ns	1.5	0.7
Ubiquitin-like 1 (sentrin) activating enzyme subunit 1 (Sae1)	AI604685	1.0	ns	1.0	ns	0.6	2.5
Proteasome beta 9 (multifunctional protease 2) (Psmb9)	AA822485	1.0	ns	0.6	5.2	0.7	0.1

FDR: false discovery rate (%), FDR > 10 = not significant (ns).
